# Case of Carinomatous Disease of Stomach

**Published:** 1847-05

**Authors:** Chas House

**Affiliations:** Springville


ART. IV.—*Case of Carinomatous disease of Stomach.*—By CHAS. HOUSE, M. D.

SPRINGVILLE, April 26, 1847.

DR. FLINT—Dear Sir :—The history of the following case, and the post mortem examination, I send you for publication, should you deem it of sufficient interest to merit an insertion in your valuable journal. The patient, Mr. L., about fifty-five years of age, referred the commencement of his disease to an injury which he received some twenty-nine years since. He came in contact with the sharp point of one of the handles of his plough while engaged in ploughing, receiving such a thrust over the pit of the stomach as to knock him down, and stun him for some time. From the effects of this accident he had but imperfectly recovered, suffering more or less from pain and tenderness in that region, when, by a striking coincidence, he suffered a similar accident, which aggravated the existing difficulty very much. Since that time he has never been exempt from a permanent soreness on pressure over the "*scrobiculus cordis*," with dyspeptic symptoms, and occasional attacks of what he called "*cramps in the stomach.*" He was never able after the last accident to do such kinds of work as required a grasping effort, such as binding grain, &c. He first came under treatment about three months ago, having slipped while carrying a pail of water, and wrenched himself slightly; he began to have burning pain, increased in paroxysms, over the abdomen, extending up to the right hypochondrium, and recurring, with slight intermissions, night and day; almost entirely precluding sleep, except during short intervals, which were spent either siting in a chair, or upon his face in bed, not being able to lie upon his back, and seldom on his side. His favourite position was upon his elbows, and buried under a profusion of blankets, so as to exclude the cold air, the contact of which, or even the hand over the abdomen, put him in great agony, such was his extreme sensitiveness. At this period, his appearance, though indicative of much suffering, did not present anything very remarkable; his pulse natural, perspiration quite profuse, and all other secretions nearly natural; yet the bowels were slightly constipated, but easily moved by a thorough cathartic. Fomentations, sinapisins, and blisters were resorted to, but without any milioration of his symptoms, which yielded only to large doses of anodynes, and that only for a short time. He was about this time subjected to the prescriptions of a '*Root Doctor*,' and when seen by me again, some four weeks after, his pulse had increased from sixty to one hundred in a minute, and he appeared haggard, and much emaciated. He now showed me, for the first time, a knotty enlargement of the lymphatics of the neck, over the left clavicle, some two inches in diameter, which he said had been forming slowly since his recent attack, but attended with no inconvenience. A large tumor, also, of much hardness, began to be distinctly definable, extending across the abdomen, and down half way from the umbilicus to the symphisis pubis. The eructations now became intensely acid, as was also the perspiration. A burning sensation in the stomach, and along the œsophagus, was incessantly complained of, and was relieved by holding cold water constantly in the mouth, By the use of antacids, this condition was alleviated for a week, when he began to vomit freely, a dark, grumous, intensely sour matter, which continued to be ejected at intervals for two days, when, completely prostrated, he sank rapidly and died; the extreme pain and soreness having ceased some hours before death.

Twelve hours after death, an examination of the bowels was made. Upon exposing the stomach, it was found enormously distended, especially in the left portion, and covered on its anterior face, and lesser curvature, from the cardia to the pylorus, by a schirrhus deposite of more than an inch in thickness. It was firmly agglutinated to the concave surface of the liver, which was perfectly healthy in appearance and structure. The stomach, with the morbid deposit and contents, nearly filled the whole abdomen, the intestines being healthy, with the exception of a few mesenteric glands, one of which was observed as large as a hickory nut, and with the same morbid degeneration. The stomach contained a large quantity of a pitchy material, extremely fœtid, as indeed was the last that was vomited during life. The mucus membrane over the diseased portion was found softened, so as easily to be scraped away, and injected with dark blood, appearing as if the blood had been effused from the whole ulcerous surface, which was eroded, and jagged in various parts. The pyloric orifice, though still pervious, was surrounded by the schirrus deposit, in common with other portions of the anterior wall, which may have been the cause of the great distension and vomiting. I regret that circumstances did not allow of a more extensive examination, and of preserving the morbid production.

Several important queries present themselves in reviewing the origin and progress of this case. Here is a disease produced by injuries received years since, and proceeding steadily to a fatal termination. Was this the result of a cancerous predisposition, or was this acquired since by an imperfect nutrition of the system, and consequent cachexia. That it was true carcinoma, and not merely chronic induration, seems proven by the morbid accumulations existing in several and non-contiguous parts of the body, as the neck, stomach, and mesenteric glands; and also from the nature of the product itself. Of its hereditary origin there is scarcely evidence, as he is the only one of seven children, some of whom are older than himself, who has died. It is true, some distant relative is remembered to have had cancer of the breast, and what person cannot exhume some distant relative, who died with what is called cancer! I have only to say in concluding this too protracted article, that if I have added any facts which may aid in elucidating this interesting class of disease, I shall feel a sincere satisfaction.

With assurances of respect,

I remain yours truly,

CHARLES HOUSE.

